# Causes of Mortality during the First Five Years of Life, Yazd City, Iran (2005-2008)

**Published:** 2014-07

**Authors:** Mohammad Reza Vafaeenasab, Nasrin Ghasemi, Samaneh Mahmoodi, Fatemeh Ghasemi

**Affiliations:** 1 Cardiovascular Research Center, Shahid Sadoughi University of Medical Sciences, Yazd, Iran;; 2 Research and Clinical Center for Infertility, Yazd Reproductive Sciences Institute, Shahid Sadoughi University of Medical Sciences, Yazd, Iran;; 3 Medical Student, Faculty of Medicine, Shahid Sadoughi University of Medical Sciences, Yazd, Iran;; 4 Deputy Health Affairs, Shahid Sadoughi University of Medical Sciences, Yazd, Iran

## Dear Editor,

Child mortality refers to deaths of children less than five years of age. According to the UNICEF reports in 2010, the number of deaths among children younger than five years old in the world was 7.6 million. Statistics for previous years (1990, 2008 and 2009) were 8.1, 8.8 and 12.4 million respectively. UNICEF reported that the top five causes of child mortality in developing countries were acute respiratory infections, diarrhea, measles, malaria, nutrition and abuse.^1^ Causes of child mortality are divided into two general categories namely; congenital and acquired. With the improved parental education, cultural transformation and better road networks, healthcare offered to children has similarly improved. However, although acquired causes of child mortality have been reduced but the congenital causes have increased. Congenital heart diseases are the cause for 50% of neonatal and 20% of child deaths.^2^

A nationwide study using data from the Child Mortality Surveillance System was carried out in Iran during 2007 to 2008 (1378-Persian calendar year). The data were acquired from the registered data across 346 District Health Networks and about 700 hospitals using Mortality Surveillance System for children under 5 years old. The most common causes of mortality were due to chromosomal abnormalities (23.4%), unintentional injuries (20.5%), respiratory diseases (9.8%), infectious disease (8%) and neurologic diseases (5.6%) whereby about 48% were girls and 52% boys.^2^ Infections and acquired diseases are the major causes of child mortality, whereas in countries with a better socioeconomic condition, congenital disorder, chromosomal defect, sudden infant death syndrome and accident are the prime causes of child death.^3^

During a descriptive cross- sectional study in the Yazd province, all 514 cases of death among children younger than 60 months of age during 2005-2008 were studied. The data were collected from those registered in the health centers of the province as well as the Vice Chancellor for Clinical Affairs of the University of Medical Sciences. The data on age, sex, cause of death and personal information which were crosschecked by a physician were documented. The data were analyzed by SPSS-15 software and the result indicated that 52.6% of mortalities were among girls and 47.4% boys. Gender difference in child deaths were not synchronized with the results of other studies since the data from a national study also indicated child mortality among girls by 48% and by 52% in boys.^2^ The result of a study conducted in 187 countries has shown that the mortality of children under 5 years was higher among boys than girls^4^ with 64.7% between 1-11 months and 35.3% between 12-60 months. A nationwide survey conducted in 2012 revealed that 62% were below 11 months and 38% were between 12 to 60 months.^2^ In a different study, 61% of deaths were between 1-11 months and 39% between 12-60 months old.^5^ The most common causes of death in our study were found to be; congenital and chromosomal malformation by 20.6% (n=116), trauma and accidents by 18.5% (n=104), heart diseases by 14.3% (n=80), respiratory diseases by 8.8% (n=50), diseases of the nervous system by 6.2% (n=35), parasitic and infectious diseases by 6.2% (n=35), endocrine and nutrition diseases by 5.3% (n=30), gastrointestinal diseases by 3.6% (n=20), prenatal diseases by 3.2% (n=17), cancers by 2.3% (n=13), diseases of the hematopoietic system by 1.3% (n=7) and urinary tract diseases by 1.3% (n=7).

Results of a meta-analysis in 2008, establish that the proportion of child mortality in trauma and accident events was 6%.^6^ Contrasting with the global and national averages,^2^ this shows the importance of accident as a critical cause of mortality in Yazd province. The data from Ruden in China during 2008 shows that the main cause of child mortality in China are pneumonia (47%), congenital abnormalities (16%), sudden death syndrome (12%), diarrhea (9%) and accidents (7%).^3^ In this study the proportion of child mortality due to genetic disorders was 51.4%.

Based on the present study, chromosomal congenital abnormalities (20.6%) are the leading cause of child mortality, which requires more attention regarding genetic consultation and prenatal care. However, the proportion of death due to accident and trauma is comparatively high which indicates the need for guiding educational programs to create parental awareness on probable dangerous behavior of children and to prevent harmful circumstances. 
